# What Is Behavioural Medicine? Commentary on Definition Proposed by Dekker, Stauder and Penedo

**DOI:** 10.1007/s12529-016-9611-6

**Published:** 2016-12-06

**Authors:** Marie Johnston, Derek Johnston

**Affiliations:** Institute of Applied Health Sciences, University of Aberdeen, Aberdeen, UK

**Keywords:** Behavioural medicine, Definition, Disciplines, Behaviour change

## Abstract

**Purpose:**

Dekker et al. (2016) propose an updated definition of behavioural medicine.

**Method:**

In this commentary, we discuss how the field and the disciplines involved have changed over time before suggesting small amendments to the proposed definition.

**Results:**

We suggest that the range of medicine which might be considered ‘behavioural’ is increasing to encompass virtually all medical practice. In addition, the role of behaviour and the potential for behaviour change as a means of improving health have become increasingly important. A defining characteristic of behavioural medicine is the involvement of multiple disciplines, working together or in parallel and, as the extent of the field expands, more disciplines are likely to be involved.

**Conclusion:**

We therefore propose that the definition should represent the full width of the research, practice and disciplines involved in behavioural medicine.

## Introduction

Dekker, Stauder and Penedo [1] propose an update of the definition and scope of behavioural medicine. Definitions of a field have value if they enable those within the field to identify with it, for example by attending its conferences and publishing its journals, and if those outside the field can recognise how it might be attractive or useful to them. In this response, we first discuss how the content and focus of behavioural medicine have been changing as a way of thinking about how the definition might change before commenting on their proposals.

## All Medical Practice Is Behavioural: the Range of Medical Practice Recognised to Be ‘Behavioural’ Is Increasing

Leaving aside the basic biomedical and pharmacological sciences, one can argue that all aspects of the ‘practice of the diagnosis, treatment and prevention of disease’ [2] involve the behaviour of many clinical and other disciplines at all stages in the process as well as the behaviour of patients and the wider population in interacting with medicine and illness. Early behavioural medicine investigations mainly concentrated on developing biofeedback interventions for diverse conditions [3] or the effect of stressors (including the onset of illness and stress-related behaviours, notably type A behaviour) on emotional and physiological responses and to a very limited extent prediction of disease. The field has expanded to include behaviour in all clinical specialties, from immunology [4] to surgery [5] as well as behaviour in the domain of public health [6]. There has been increasing focus on the behaviour of those delivering healthcare with greater recognition that their behaviour may have important influences on health outcomes [7–9] and that delivering healthcare may influence the health outcomes of the professionals themselves [10].

The importance of avoiding ‘unhealthy medicine’ [11] by ensuring that evidence is implemented in practice and that we are not ‘all breakthrough, no follow through’, more work is being done on knowledge transfer into practice with journals such as *Implementation Science* (http://implementationscience.biomedcentral.com/ ) and *Translational Behavioral Medicine* (http://www.springer.com/medicine/journal/13142) representing these additional fields. In a systematic review of intervention frameworks, Michie et al. [12] noted the range of function and policies engaged in behavioural interventions and, by implication, that the range of relevant authorities goes well beyond medicine, including educational, fiscal, environmental and planning, whose behaviour influences health outcomes.

It is essential therefore that the definition and scope of behavioural medicine are inclusive with respect to the gamut of medical practice involved.

## There Is More ‘Behaviour’ in Behavioural Medicine

Since the original definition, there has been an increasing focus on behaviour as a cause and consequence of health status, to complement the earlier emphasis on stress, emotions, beliefs, traits and mental health. The Decade of Behavior from 2000 to 2010 (http://www.asanet.org/footnotes/nov00/indextwo.html ) was a response to the increasing recognition of the role of behaviour in addressing important societal challenges including health and has been accompanied by an upsurge in research and practice activity related to behaviour.

**Fig. 1:**
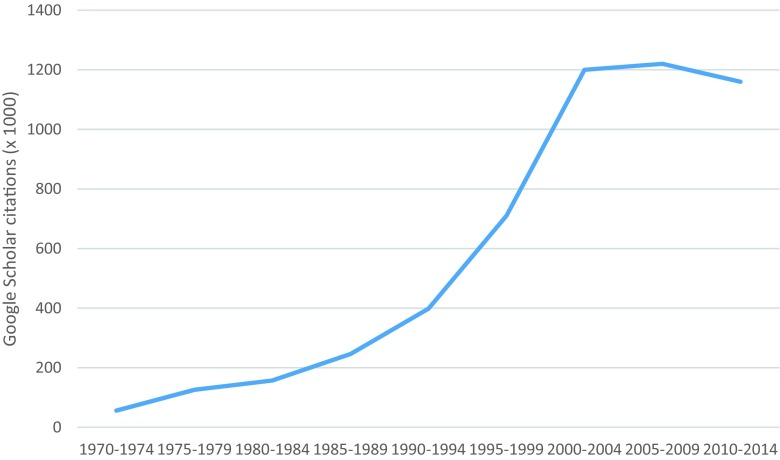
‘Behaviour change’ citations in Google Scholar from 1970 to 2014

In the 1970s, behavioural medicine research and practice developed in two main domains: the first largely laboratory studies of the effects of stress and coping on physiological processes, and the second centred on psychosocial processes (roughly translated as the interaction between psychic and social factors) such as stress, emotions and personality along with socio-demographic and environmental factors investigated as determinants of health outcomes and, where modifiable, as opportunities for improving health in individuals and populations. Gradually, behaviour per se has become more important not only as a key mediator of the relationship between psychosocial processes and health but also as a direct cause of illness or good health and as a target for intervention at population, community and individual levels. Publications on ‘behaviour change’ have increased dramatically since the 1970s (see Fig. 1) and have become a priority for government policy and for clinical and public health services in many countries and are illustrated here by the UK Government advisory documents [13–15].

## How Have the Disciplines Changed?

The 1970s and 1980s saw the increasing interest of the mental health disciplines in somatic health and the public health disciplines in psychological, behavioural and social influences on health. New sub-disciplines were emerging as were collaborative approaches across disciplines. In psychology, there was a debate about how to subdivide and label the field, exemplified in the UK by correspondence about the possible labels for the subdivisions of psychology, including medical, health, behavioural health, public health, clinical and clinical health.

**Fig. 2:**
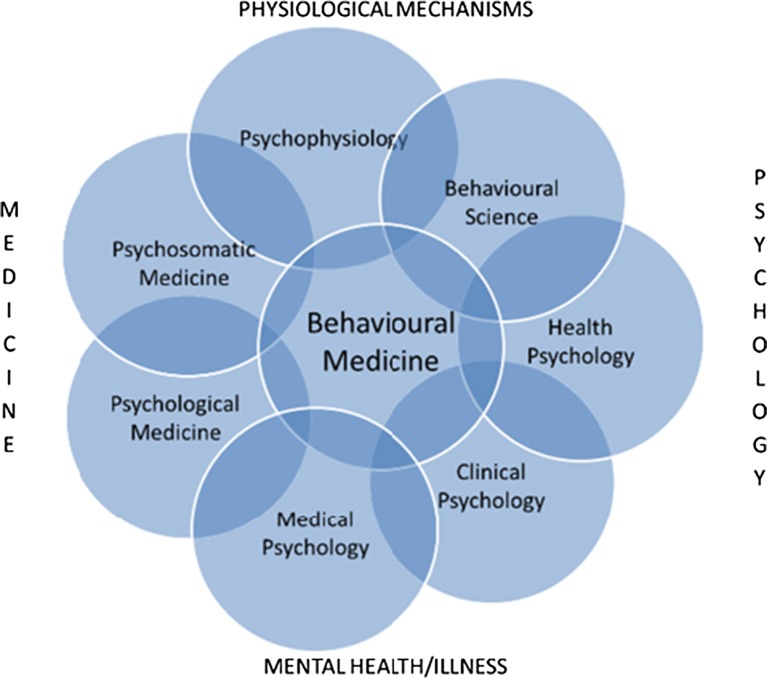
Overlaps between psychological and other disciplines in behavioural medicine

In Fig. 2, we sketch the overlaps between the psychology sub-disciplines and the related disciplines that involve multiple disciplines. Similar diagrams could be drawn for each discipline involved in behavioural medicine. We suggest two main points. First is that there are no clear lines between disciplines and sub-disciplines but rather that they merge into each other. This seems entirely appropriate for scientific development and is a pattern repeated in other research fields. Nevertheless, it may create difficulties for employment in practical applications where posts are advertised by discipline, but not if they are defined by competences. Scottish Government developed a competency framework to ensure that behaviour change interventions could be delivered by the people with competence rather than a disciplinary badge [15]. Second, each of the sub-disciplines of psychology has its own relationship with the multi-disciplinary disciplines and is therefore not entirely interchangeable.

However, in addition, these subdivisions of psychology have become closer to other mono-disciplines as they engage in the multi-disciplinary process of behavioural medicine. We are more likely to collaborate in research with other social sciences such as sociology, geography and economics and other medical sciences such as immunology, cardiology and genetics. These collaborations involve not only meeting with other disciplines but also co-designing projects, reading each other’s literature, using methods from other disciplines that complement or improve those of our own discipline etc. It is our impression that the same would be true for other disciplines involved in behavioural medicine, i.e. that our work in the domain of behavioural medicine has enabled closer collaborations between disciplines.

There is still a question of how we describe this collaboration between disciplines and the vexed question of inter-versus multi-disciplinarity. Choi and Pak [16] define the input of different disciplines to be separate but additive in multi-disciplinarity but interactive and integrative in interdisciplinary work. Neither of these definitions fully encompasses the variety of working collaborations in behavioural medicine as both formats contribute to the field. Since there is uncertainty about the meaning of these terms, it would surely be wiser to refer to ‘multiple disciplines’ leaving open the range of possible working relationships.

## The Proposed New Definition and Scope

The changes over time since the ISBM Charter have largely been changes in the intensity of effort and in fulfilling the full scope of the field. Behavioural medicine research and practice have expanded their scope, recognising that behavioural factors influence the full range of health and healthcare outcomes. At the same time, there has been an increasing emphasis on behaviour to complement other psychological processes in both policy and research.

These aspects of the definition require no updating but the proposed expanded version is successful in making the extent of the field more transparent.

However, it is not clear why some elements of the definition have been lost e.g. why should ‘etiology’ be omitted when there continues to be investigation of etiology and not simply as prevention and health promotion. In listing the biobehavioural mechanisms, it is surely essential to include behavioural processes. Finally, we consider that it is important to fully represent the nature of the involvement of the *many* behavioural medicine disciplines.

We therefore propose these minor amendments (highlighted) to Dekker et al.’s proposal:

*Behavioral medicine can be defined* ***as the field of research involving multiple disciplines*** *concerned with the development and integration of biomedical and behavioral knowledge relevant to physical health and disease, and the application of this knowledge to* ***etiology*** *, prevention, health promotion, diagnosis, treatment, rehabilitation, and care. The scope of behavioral medicine extends from* ***fundamental*** *biobehavioral mechanisms (i.e. the interaction of biomedical processes with psychological,* ***behavioral*** *, social, societal, cultural and environmental processes),* ***to behavioral processes*** *in clinical diagnosis and intervention, and in public health.*
